# NAFLD and Atherosclerosis Are Prevented by a Natural Dietary Supplement Containing Curcumin, Silymarin, Guggul, Chlorogenic Acid and Inulin in Mice Fed a High-Fat Diet

**DOI:** 10.3390/nu9050492

**Published:** 2017-05-13

**Authors:** Antonella Amato, Gaetano-Felice Caldara, Domenico Nuzzo, Sara Baldassano, Pasquale Picone, Manfredi Rizzo, Flavia Mulè, Marta Di Carlo

**Affiliations:** 1 Biological, Chemical and Pharmaceutical Sciences and Technologies (STEBICEF), University of Palermo, Palermo 90128, Italy; gaetanofelice.caldara@unipa.it (G.-F.C.); sara.baldassano@unipa.it (S.B.); flavia.mule@unipa.it (F.M.); 2 Institute of Biomedicine and Molecular Immunology “Alberto Monroy” (IBIM), Consiglio Nazionale delle Ricerche (CNR), 90146 Palermo, Italy; domenico.nuzzo@ibim.cnr.it (D.N.); pasquale.picone@ibim.cnr.it (P.P.); 3 Biomedical Department of Internal Medicine and Medical Specialties, University of Palermo, Palermo 90127, Italy; manfredi.rizzo@unipa.it

**Keywords:** non-alcoholic fatty liver disease, atherogenic lesions, diet-induced obesity, natural dietary supplement, renin-angiotensin system imbalance, Profiler PCR array

## Abstract

Non-alcoholic fatty liver disease (NAFLD) confers an increased risk of cardiovascular diseases. NAFDL is associated with atherogenic dyslipidemia, inflammation and renin-angiotensin system (RAS) imbalance, which in turn lead to atherosclerotic lesions. In the present study, the impact of a natural dietary supplement (NDS) containing *Curcuma longa*, silymarin, guggul, chlorogenic acid and inulin on NAFLD and atherosclerosis was evaluated, and the mechanism of action was examined. C57BL/6 mice were fed an HFD for 16 weeks; half of the mice were simultaneously treated with a daily oral administration (os) of the NDS. NAFLD and atherogenic lesions in aorta and carotid artery (histological analysis), hepatic expression of genes involved in the NAFLD (PCR array), hepatic angiotensinogen (AGT) and AT_1_R mRNA expression (real-time PCR) and plasma angiotensin (ANG)-II levels (ELISA) were evaluated. In the NDS group, steatosis, aortic lesions or carotid artery thickening was not observed. PCR array showed upregulation of some genes involved in lipid metabolism and anti-inflammatory activity (Cpt2, Ifng) and downregulation of some genes involved in pro-inflammatory response and in free fatty acid up-take (Fabp5, Socs3). Hepatic AGT, AT_1_R mRNA and ANG II plasma levels were significantly lower with respect to the untreated-group. Furthermore, NDS inhibited the dyslipidemia observed in the untreated animals. Altogether, these results suggest that NDS prevents NAFLD and atherogenesis by modulating the expression of different genes involved in NAFLD and avoiding RAS imbalance.

## 1. Introduction

Non-alcoholic fatty liver disease (NAFLD) is the most frequent hepatic disorder in developed countries and may lead to steatohepatitis, cirrhosis and liver cancer. NAFLD is also considered the hepatic component of metabolic syndrome (MetS) because it is associated with atherogenic dyslipidemia, obesity and type 2 diabetes (T2DM) [1]. The precise mechanism of the onset and progression of NAFLD remains unclear although increased fatty acid syntheses, oxidative stress and inflammation may play a fundamental role [2]. 

Emerging evidence suggests that angiotensin (ANG) II, a pro-oxidant cytokine, synthesized mainly from the hepatic precursor angiotensinogen (AGT), may have a relevant importance in the pathogenesis of NAFLD by generating reactive oxygen species and regulating the production of pro-inflammatory mediators [3]. Patients with NAFLD present elevated ANG II levels [4,5], and animals with liver steatosis show increased hepatic expression of AGT, AGT II and ANG II type 1 receptor (AT_1_R) [6,7]. The renin-angiotensin system (RAS) and its primary mediator ANG II have also a direct influence on the progression of the atherosclerotic process via effects on endothelial function, inflammation, fibrinolytic balance and plaque stability [8]. Increasing clinical evidence supports a strong association between NAFLD and cardiovascular diseases (CVD), which represents the principal cause of death in NAFLD patients, more so than liver-related complications [9,10]. Patients with NAFLD have an altered flow-mediated vasodilatation and increased carotid-artery intimal medial thickness, two reliable markers of subclinical atherosclerosis [11]. Therefore, the involvement of AGT II in NAFLD pathogenesis and in atherosclerotic plaque formation may provide one of the possible links between NAFLD and accelerated atherogenesis. Accordingly, the use of RAS blockers seems to be potentially useful as a therapeutic approach against NAFLD [12] and atherosclerosis [13]. To date, there is no single approved pharmacologic therapy to treat metabolic dysfunctions occurring in obesity, such as NAFLD and related atherosclerosis. The backbone of therapy currently includes lifestyle management to induce weight loss and therapeutic treatment to reduce cardiovascular risk or hyperglycemia [14]. Recently, natural herbs have been the focus of many researches both because of their safety and efficacy and because their potential bio-active ingredients could help to prevent or treat obesity and the related metabolic disorders [15]. The natural dietary supplement (NDS) used in this study contains extracts from *Cynara scolymus* (chlorogenic acid), *Silybum marianum* (silymarin), *Taraxacum officinale* (inulin), *Curcuma Longa* (curcuma) and *Commiphora mukul* (guggul), plant extracts that exert protective actions mainly towards the liver. Recent evidence has shown that the treatment with the NDS exerts beneficial effects in patients with MetS, reducing anthropometric parameters and total cholesterol levels, but the mechanism of action is still unknown [16]. 

This study aims to investigate whether the treatment with this NDS is able to prevent the development of NAFLD and related atherosclerotic lesions in aorta and carotid artery in a mouse model of diet-induced obesity (DIO). In order to investigate the mechanism of action of the natural supplement, its ability to modulate the expression of some RAS components (AGT and AT_1_R mRNA in liver and circulating concentration of AGT II) or of genes involved in NAFLD was examined. In addition, the impact of the NDS treatment in the plasma lipid profile was also analyzed.

## 2. Materials and Methods 

### 2.1. Animals 

The procedures were performed in accordance with the conventional guidelines for animal experimentation (Italian D.L. (Legislative Decree) No. 116 of 27 January 1992 and subsequent variations) and the recommendations of the European Economic Community (86/609/ECC).

Male C57BL/6J (B6) mice, purchased from Harlan Laboratories (San Pietro al Natisone Udine, Italy) at 4 weeks of age, were housed in a room with controlled temperature and dark-light cycles, with free access to water and food. After acclimatization (1 week), the animals were weighed and divided into two groups, both fed a high-fat diet (HFD) (PF4051/D, Mucedola, Milan, Italy) composed of 60% of energy as fat, 20% protein and 20% carbohydrates, for 16 weeks.

It has been shown that these animals, consequent to an HFD, develop obesity, hyperglycemia [17], hepatic steatosis [18], atherosclerosis [19] and neurodegeneration [20].

One group served as a control of obesity-related dysfunctions (*n* = 6, untreated group), and the other one (*n* = 6, treated group) received, simultaneously to the HFD, a daily administration of NDS (0.9 mg/mouse) for 16 weeks. The dose given to the DIO mice was extrapolated from the human dosage (1.6 g/day) and calculated on the basis of the average body weight (40 mg). 

The NDS used in this study is, in Italy, commercialized under the name Kèpar^®^ and was provided by Rikrea^®^ S.r.l. (Modica-RG, Italy). The main constituents of the NDS are plant-derived polyphenolic compounds that are well known for their antioxidant and anti-inflammatory properties. In particular, NDS consists of extract from five plant sources, and each extract was obtained from a different part of the plant (Table 1). 

The tablets of the NDS (Kèpar, Batch No. SL0010) were ground by pestle and 9 mg of powder dissolved in 200 µL of water and used as stock solution. The daily dose was freshly made up, by diluting 1:10 the stock solution (0.9 mg/mouse in 20 µL), and was administered by oral gavaging. The administrated dose contained: 0.09 mg of *Curcuma*, 0.057 mg of silymarin, 0.0135 mg of guggul lipids, 0.008 of chlorogenic acid and 0.002 mg of inulin. During the 16 weeks of the treatment, changes in body weight and food-intake, determined by measuring the difference between the pre-weighed chow and the weight of chow at intervals of 24 h [21], were periodically monitored and compared between the two groups of animals. After 16 weeks of treatment, mice were sacrificed, and blood, liver, aorta and carotid artery were immediately collected for subsequent analysis.

### 2.2. Histological Analysis

Aorta, carotid arteries and liver specimens excised from each hepatic lobe were fixed in 4% formalin for 24 h. After this treatment, the tissues were dehydrated in alcohol and embedded in paraffin wax. Paraffin histological sections (5 μm thick) were stained with hematoxylin and eosin and observed using an automated Leica DM5000 B microscope (Leica, Milan, Italy) connected to a high-resolution camera, Leica DC300 F (Leica, Milan, Italy). According to the Non-alcoholic Steatohepatitis Clinical Research Network (NASH CRN) scoring system, steatosis was determined by analyzing the morphology and percentage of lipid vesicles in hepatocytes [22]. 

### 2.3. Atherogenic Index (AIS) 

The atherogenic index serum (AIS), which is the measure of the atherosclerotic lesion extent based on serum lipids [23], was determined in all groups. The atherogenic index serum is calculated using the formula AIS = total cholesterol (TC)/HDL [24]. 

### 2.4. Biochemical Analysis

Lipid profile was measured in mice fasted for 6 h with free access to water. After this time, the mice were euthanized, and the blood was drawn by cardiac puncture and immediately transferred into chilled tubes containing a final concentration of 1 mg/mL EDTA. Then, the samples were centrifuged at 825 g for 10 min, and the obtained plasma was stored at −80 °C until analysis. Plasma triglyceride, cholesterol, low density lipoprotein (LDL), high density lipoprotein (HDL) levels and AST- and ALT-serum concentrations were measured using the ILAB 600 Analyzer (Instrumentation Laboratory, Bedford, Massachusetts). 

### 2.5. Quantitative Real-Time qPCR

Total RNA from livers of treated and untreated obese mice were extracted using the RNEasy Mini Kit (Qiagen, Milan, Italy). Two nanograms of RNA were used to synthesize the first strand cDNA using the RT First-Strand kit (Qiagen, Milan, Italy). Synthesized cDNAs were amplified using RT2 SYBR Green/ROX qPCR Mastermix (Qiagen, Milan, Italy) and StepOne Real-Time instrument (Applied Biosystem, Foster City, CA, USA). 

Gene expression analysis was performed using sequence primers for mice AGT, AT_1_R and β-actin (SigmaLife Sciences, Milan, Italy). The primers were as follows: AGT forward 5’-GTA CAG ACA GCA CCC TAC TT-3’, reverse 5’-TTG TTG AAG AGG CAC TGC AC-3’; AT_1_R forward 5’-GAC CAA CTC AAC CCA GAA AAGC-3’, reverse 5’-CCT TTG TCG AAC CAC CACTA-3’; β-actin forward 5’-CGG GAT CCC CGC CCT AGG CAC CAG GGT-3’, reverse 5’-GGA ATT CGG CTG GGG TGT TGA AGG TCT CAAA-3’. Each PCR reaction was amplified in triplicate, and levels of expression were calculated after normalization to β-actin. On the basis of the Ct value (threshold cycle; the number of reaction cycles after which fluorescence exceeds the defined threshold) of the examined gene and of the internal control gene, the relative expression level of RNA was calculated according to the 2^−ΔΔCt^ approximation method. 

### 2.6. RT^2^ Profiler PCR Array

Synthesized cDNAs from NDS-treated and NSD-untreated livers were added to 96-well reaction plates of the Mouse Fatty Liver PCR Array (PAMM-157Z, SABiosciences, Qiagen, Milan, Italy) according to the manufacturer's instructions. The array profiles the expression of 84 key genes involved in the mechanisms of non-alcoholic fatty liver disease (NAFLD) and hepatic insulin resistance. The reaction was performed by using a StepOne Real-Time instrument (Applied Biosystem, Foster City, CA, USA). Analysis was performed using the spreadsheet provided by Qiagen Company, Milan, Italy.

### 2.7. Measurement of Circulating Levels of Angiotensin II

Quantification of plasma AGT II was carried out by the ELISA kit for mice (Enzo Life Sciences, Inc. Farmingdale NY, USA) according to the manufacturer’s instructions. The experimental detection limit of the analysis was 3.9 pg/mL.

### 2.8. Statistical Analyses

Results are shown as means ± the standard error of the mean (S.E.M.). The letter n indicates the number of animals. Statistical analyses were performed using Prism Version 6.0 Software (Graph Pad Software, Inc., San Diego, CA, USA). The comparison between the groups was performed by ANOVA followed by Bonferroni’s post-test. A *p*-value ≤ 0.05 was considered statistically significant. 

## 3. Results

### 3.1. The Natural Dietary Supplement Prevents the Development of NAFLD

**Figure 1:**
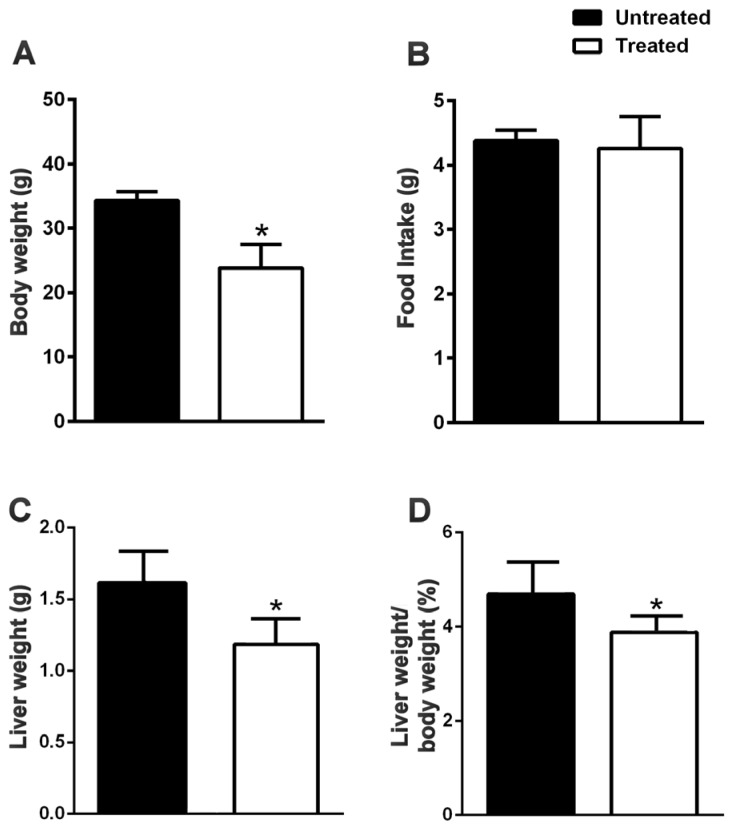
NDS reduces food intake, body and liver weight in HFD mice. Effects of the NDS treatment (0.9 mg/mouse) on body weight (**A**), food intake (**B**), liver weight (**C**) and the ratio of liver weight/body weight (**D**), in mice fed a high-fat diet (HFD). Data are the means ± S.E.M. (*n* = 6/group). * *p* ≤ 0.05.

At the end of the treatment, the untreated mice had greater mass gain compared to the animals treated with NDS (Figure 1A), although no difference in the food intake was observed (Figure 1B). Furthermore, the liver weight (Figure 1C) and ratio liver weight/body weight (Figure 1D) was higher than the treated group. In the untreated mice, steatosis affected the liver gross appearance. 

**Figure 2:**
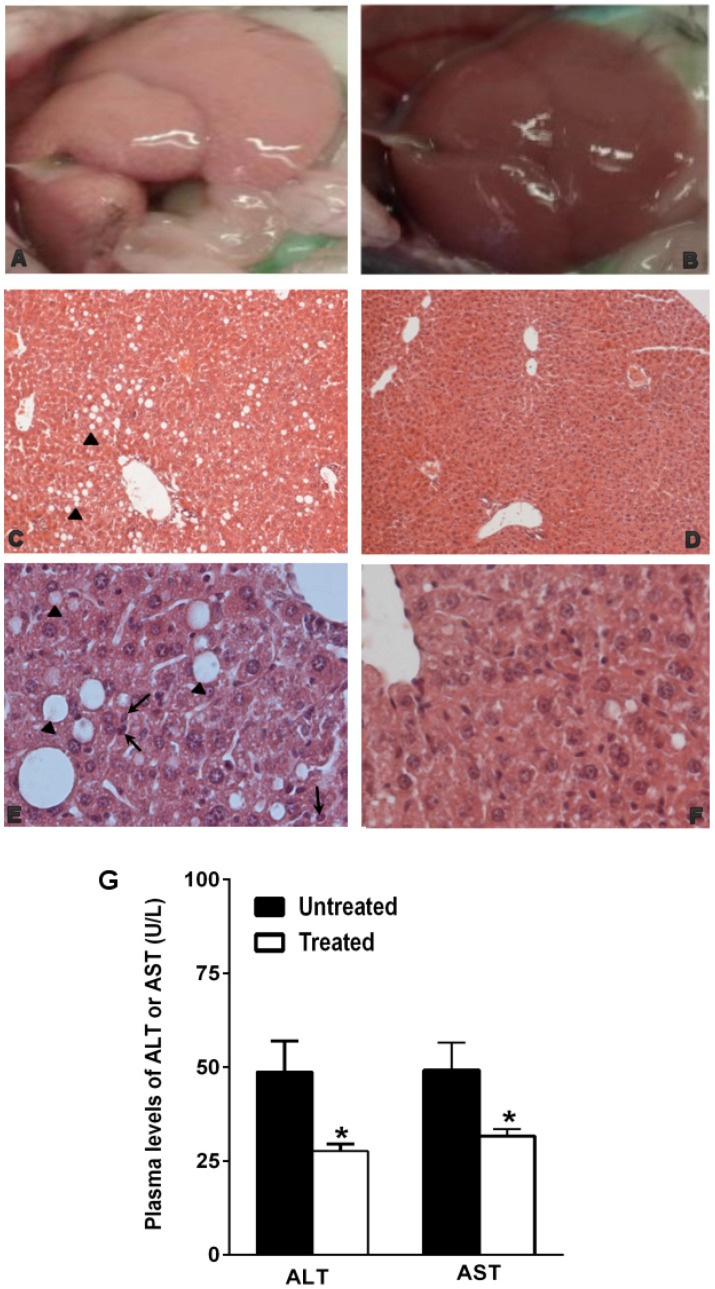
NDS prevents NAFLD development in diet-induced obesity (DIO) animals. Representative images of gross anatomy and histological cross-sections of liver from NDS-treated and untreated high-fat diet (HFD) mice. Liver morphologies of untreated HFD mice (**A**) and NDS-treated HFD mice (**B**). Cross-sections from untreated HFD mice (**C**,**E**) show micro- and macro-vesicular steatosis (black arrowhead) with polymorphonuclear cell infiltration (black arrow). Histological cross-sections from NDS-treated HFD mice (**D**,**F**) show normal hepatocytes and maintaining of the lobular architecture. Hematoxylin and eosin stain. Original magnification: (C,D) = ×200, (E,F) = ×400. (**G**) Plasma levels of AST and ALT. Data are the mean values ± S.E.M. (*n* = 6/group). * *p* ≤ 0.05.

In particular, the organ appeared enlarged with rounded edges. Moreover, it was pale-yellowish color, friable with a greasy texture attributable to the fat accumulation in the hepatic parenchyma (Figure 2A). Histologically, the liver exhibited micro- and macro-vesicular steatosis (Grade I: >5%–33%) in the perivenular area (Zone 3) and transition area (Zone 2) (Figure 2C) with focal infiltration of polymorphonuclear cells (Figure 2E). The hepatocytes showed a typical foamy aspect; numerous small vacuoles coalesced and created cleared space displacing the nucleus to the periphery of the cells (Figure 2E). On the contrary, in the NDS-treated mice, the liver gross anatomy was not affected by fat accumulation (Figure 2B), and the histological analysis did not reveal the presence of steatosis (Grade 0: <5%), but only the presence of small inclusions of lipids within hepatocytes (Figure 2D–F). Moreover, in agreement with the liver maintaining the lobular architecture, plasma AST and ALT were significantly lower in treated mice than in the untreated ones (Figure 2G). 

**Figure 3:**
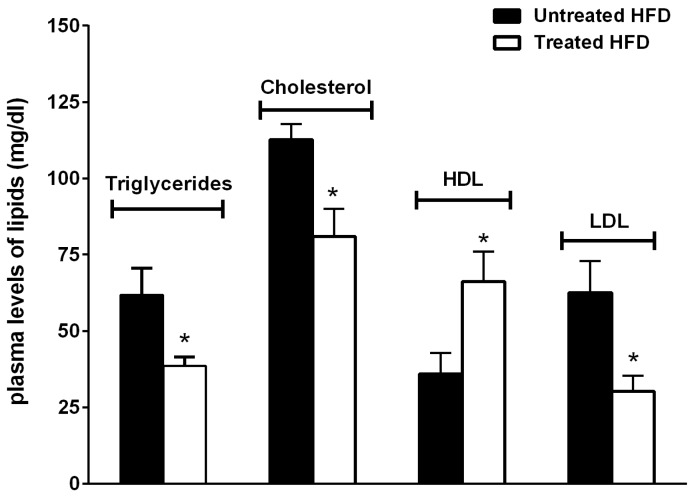
NDS prevents altered the plasma lipid profile. Effects of NDS treatment (0.9 mg/mouse) on plasma lipid concentrations. Data are the mean values ± S.E.M. (*n* = 6/group).* *p* ≤ 0.05.

In addition, NDS-treated mice showed decreased triglycerides, cholesterol and LDL and increased HDL plasma levels in comparison with untreated animals (Figure 3). 

### 3.2. The Natural Dietary Supplement Modulates the Expression of Genes Involved in NAFLD

**Figure 4:**
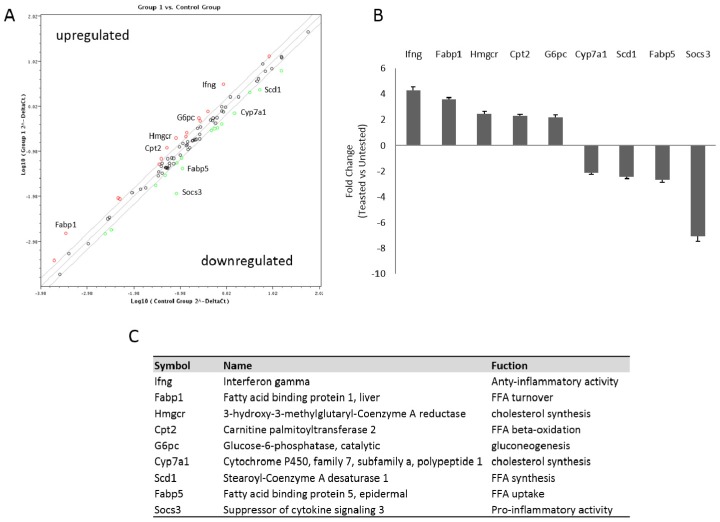
NDS reduces the expression of genes involved in NAFLD pathogenesis. Livers of untreated and treated high-fat diet (HFD) mice were used to perform the RNA for the PCR array analysis. (**A**) Scatter plot of relative expression levels for each gene in mice samples (treated vs. untreated). The figure depicts a log transformation plot of the relative expression level of each gene (2^−ΔCt^) between untreated (*x*-axis) and treated mice (*y*-axis). The grey lines indicate a two-fold change in gene expression threshold. Red rings indicate upregulated genes; green rings indicate downregulated genes. (**B**) Histogram of some up- and down-regulated genes with a greater than two-fold expression change. (**C**) Table of the names and functions of the quantitative real-time PCR of the chosen genes.

To compare differences in the profile gene expression involved in NAFLD between the treated and the untreated groups, we analyzed hepatic expression levels of 84 genes by PCR array analysis. The position of the genes is signified in Table S1. In the hepatic tissue of treated mice, the expression levels of 23 genes were affected (Table S1). Among these, nine were up- or down-regulated by more than two-fold in comparison with the untreated animals (Figure 4A–C). In particular, NDS treatment upregulates genes involved in lipid metabolism and anti-inflammatory mediators (Cpt2, Ifng), whereas it downregulates genes involved in FFA up-take and in pro-inflammatory activity (Fabp5, Socs3).

### 3.3. The Natural Dietary Supplement Prevents Atherosclerosis Development 

**Figure 5:**
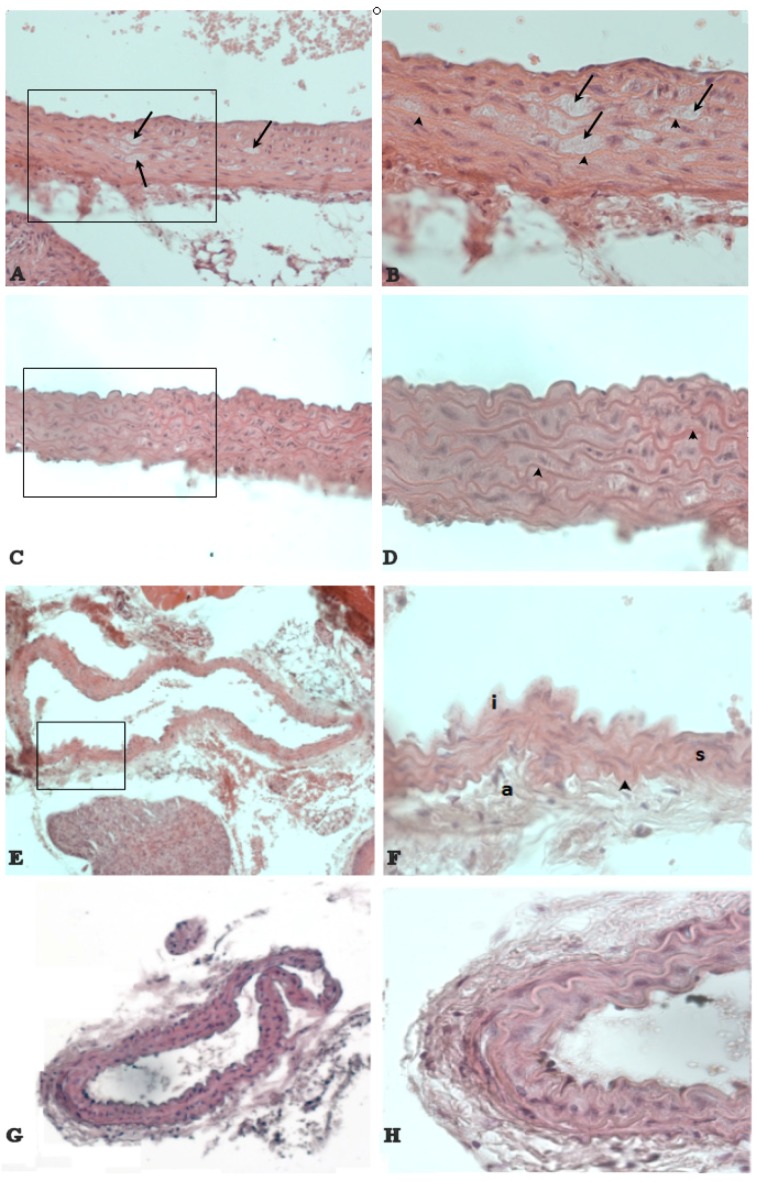
NDS prevents the development of atherosclerotic lesions. Representative images of histological cross-sections of aortic arch and carotid artery in high-fat diet (HFD) mice. The black arrow (**A**,**B**) denotes the prominent fatty streak between elastic laminae (arrowhead) in the aortic root wall of untreated vs. treated (**C**,**D**) HFD mice. Carotid artery shows a hyperplasia of the intima (i) in untreated HFD mice (**E**,**F**). On the contrary, no alterations of carotid artery are detected in treated HFD mice (**G**,**H**). a = tunica adventitia. s = smooth muscle cells. Hematoxylin and eosin stain. Original magnification: (A,C) = ×400; (B,D,F) and (H) = ×600; (E,G) = ×200.

Untreated mice showed atherosclerotic lesions with features of the earliest stages of the disease. In fact, the observed lesions did not develop beyond the fatty-streak presence. The lesions of aorta were confined to the aortic root and were characterized predominantly by lipid-laden areas in the tunica media, between smooth muscle cells and elastic lamina (Figure 5A,B). Carotid arteries showed an increased thickening of the intima characterized by hyperplasia and the presence of myocytes proliferating from the tunica media (Figure 5E,F). On the contrary, in NDS-treated mice, neither lesions in aortic root (Figure 5C,D), nor carotid intimal hyperplasia and alterations in the tunica media or adventitia (Figure 5G,H) were observed.

**Figure 6:**
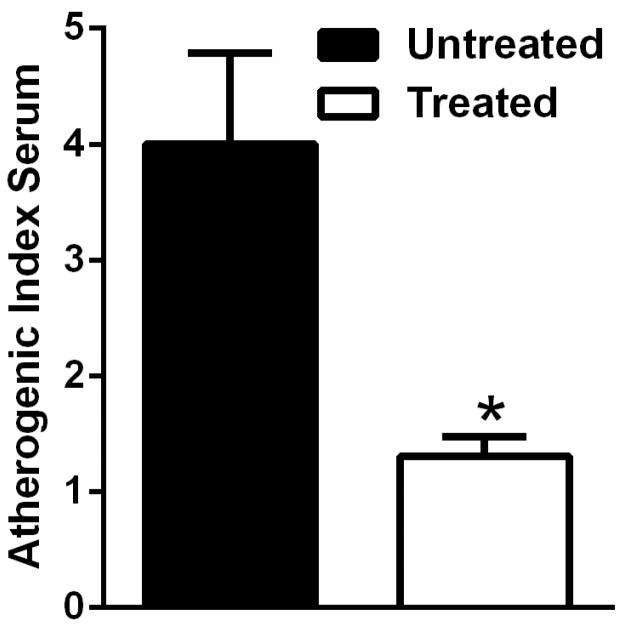
Atherogenic index serum (AIS) in HFD mice is reduced by NDS. Effects of NDS treatment (0.9 mg/mouse) on AIS of high-fat diet (HFD)-fed mice. Data are the mean values ± S.E.M. (*n* = 6/group). * *p* ≤ 0.05.

According to these data, the AIS was significantly higher in untreated HFD-fed mice in comparison with the treated obese mice (Figure 6).

### 3.4. The Natural Dietary Supplement Reduces RAS Component Expression 

**Figure 7:**
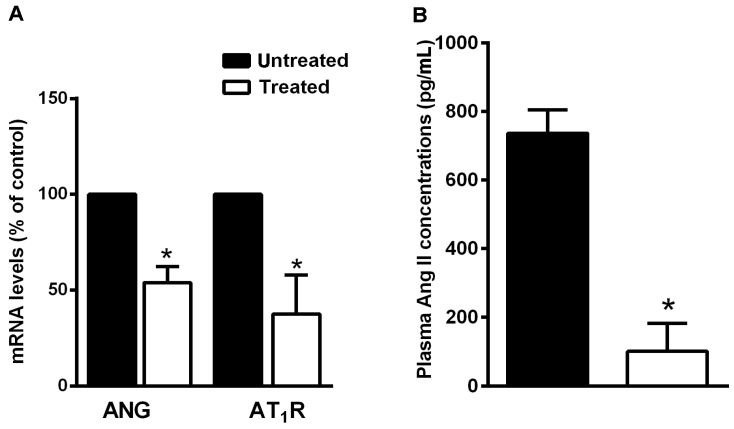
NDS reduces RAS component expression. (**A**) Expression levels of AGT and AT1R mRNAs in liver of treated high-fat diet (HFD) mice and the untreated HFD group, by real-time RT-PCR analysis. Data are the mean values ± S.E.M. (*n* = 3/group). * *p* ≤ 0.05. (**B**) Effects of NDS treatment (0.9 mg/mouse) on circulating levels of angiotensin II in HFD mice, by ELISA. Data are the mean values ± S.E.M. (*n* = 6/group). * *p* ≤ 0.05.

To investigate whether variations in the levels of the RAS system are specifically involved in the preventive effects of the natural supplement, we analyzed the hepatic mRNA levels of AGT and AT1R by quantitative real-time PCR and circulating ANG II concentration by ELISA assay. In NDS-treated mice, hepatic AGT and AT1R mRNA expression and plasma ANG II levels were significantly lower in comparison with untreated mice (Figure 7A,B).

## 4. Discussion

The present study shows that the natural supplement, here utilized, is able to prevent the development of NAFLD and atherogenic lesions in HFD obese mice. Such a preventive role is determined by its ability to reduce the expression of the RAS components (AGT, AT1R and AGT II) and modulate positively the expression of genes involved in NAFLD. The NDS also improves the lipid profile, a typical obesity-related dysfunction. 

In recent years, there has been an increasing interest in the use of plant extracts as potential therapeutic agents. A mixture of natural products is used in various therapeutic areas obtaining a number of interesting outcomes due to their synergistic effects [25]. The natural dietary supplement used in this work, known as Képar, is used in the Italian market to treat liver discomfort, caused by gallstones, cirrhosis and toxic agents. It is composed of several plant extracts (*Curcuma*, silymarin, guggul, chlorogenic acid and inulin) that exert, at least individually, beneficial effects on different components of MetS. Curcumin improves insulin resistance and dyslipidemia [26]; silymarin exerts anti-inflammatory effects in animal models of NAFLD [27]; guggul lipids have been successfully used in obesity and hypercholesterolemia [28]; and chlorogenic acid, as well as inulin, may improve lipidic and glycidic metabolism [29,30]. In the present study, we have demonstrated that the diet natural supplement is able to prevent body weight gain, hepatic fat accumulation, atherosclerotic lesions development and dysregulation of lipidic metabolism. In fact, NDS-treated HFD mice showed a body weight significantly lower than the untreated HFD animals, without any differences in the food intake. This suggests that NDS prevents body mass gain by an independent mechanism from central control of the feeding behavior. 

It is interesting to note that our study represents the first experimental evidence for a preventive role of the natural supplement against obesity-associated steatosis development. In fact, the liver of treated obese mice did not show hepatomegaly or other histomorphological alterations; on the contrary, the untreated group liver showed the presence of moderate micro- and macro-vesicular steatosis in Zone 3, a zonal distribution highly associated with the severity of steatosis [31]. The positive impact of NDS treatment, besides liver morphology, was also observed on the hepatic function, as suggested by the reduced ALT and AST plasma levels in treated-obese animals compared to the untreated group. In addition, the results showed that plasma triglycerides and LDL were lower and HDL higher in treated HFD animals in comparison to the untreated mice, demonstrating that NDS exerts beneficial effects on lipid metabolism and, consequently, on cardiovascular functions [32]. 

In order to examine the mechanism by which NDS prevents NAFLD, we also analyzed and compared the hepatic expression of genes involved in NAFLD pathogenesis in treated vs. untreated animals. For the first time, it was demonstrated that NDS is able to modulate different signaling pathways involved in de novo hepatic lipogenesis, lipid oxidation and inflammatory responses. In fact, in NDS-treated liver, genes involved in fatty acid turnover (such as Fabp1 and Cpt2) and anti-inflammatory activity (Ifng) were upregulated, while genes involved in FFA uptake (Fabp5), lipogenesis (Scd1) and inflammation (Socs3) were downregulated. The increased level of Cpt-2 and Fabp-1 provides an explanation of the reduced hepatic lipid depots observed in the NDS-treated liver. Cpt-2 is involved in beta-oxidation and Fabp-1 in the rapid removal of fatty acid in the oxidative organelles [33]. Downregulation of Fabp5 and Scd1 also contributes to preventing hepatic lipid accumulation, as Fabp5 leads to the fatty acid uptake [34] and Scd1 converts saturated FA to monounsaturated FA, the major substrates necessary for the synthesis of other lipids [35]. On the other hand, Scd1 knockout mice result in being resistant to the development of obesity and hepatic steatosis [36], and the fatty livers of ob/ob mice show increased Scd1 expression [37]. 

Microarray analysis also showed that the treatment with the natural supplement is able to modulate the expression of factors involved in the inflammatory process. In particular, the liver of treated HFD mice showed upregulation of Ifng, a protective mediator against the liver inflammatory process [38], and a very strong downregulation of Socs3, usually overexpressed in inflamed steatotic liver [39], providing a molecular basis for the protective role of the NDS in the HFD liver. 

It is well known that NAFLD shares many risk factors with cardiovascular diseases, implying a close relationship between NAFLD and adverse cardiovascular events, such as hypertension and atherosclerosis [9]. Accordingly, we analyzed the impact of NDS treatment on the development of atherogenic lesions in our animal models. Our results showed that no atherosclerotic lesions were present in the vessels of NDS-treated obese mice. On the contrary, early hallmarks of atherosclerosis were highlighted in the untreated-HFD group. In fact, in agreement with Whitman’s report [40], we observed “fatty streak-type” lesions in the aortic root, representative of foam cells, which are lipid-laden macrophages. We also observed carotid artery intimal-medial wall thickening. Additionally, AIS, a measure of the atherosclerotic lesion extent based on serum lipid concentration [23,24], was significantly lower in NDS-treated animals compared with untreated mice, suggesting a preventive action of the natural supplement on atherogenesis development. Therefore, the improvement of the lipid profile could explain the absence of lesions in the NDS-treated animals. Different mechanisms explain the increased risk of cardiovascular events in patients with NAFLD. In fact, besides the proatherogenic lipid profile, the disease is associated with an increased production of pro-inflammatory cytokines [41,42] and RAS imbalance. Patients with NAFLD present elevated circulating levels of ANG II and over-activation of intrahepatic RAS [4,5]. Our results showed that in the liver of NDS-treated obese mice, angiotensinogen and AT_1_R mRNA were significantly decreased, as well as the circulating levels of ANG II, suggesting that the treatment with the natural supplement protects from NAFLD and atherogenesis by preventing RAS imbalance. 

## 5. Conclusions

In conclusion, the natural dietary supplement (Kèpar) is effective in protecting against the development of NAFLD and atherosclerotic lesions in obesity conditions. The NDS prevents liver fat accumulation, development of atherosclerotic lesions and improves hyperlipidemia. These beneficial effects seem to be mediated by the ability of the natural supplement to modulate the expression of different genes involved in NAFLD and to prevent the imbalance of RAS components.

## Figures and Tables

**Table 1:** Ingredients of the natural dietary supplement (NDS) formulation.

Herbal Components	Part Used	Quantity/100 g
*Cynara scolymus*, e.s. tit. 2.5% chlorogenic acid	Leaf	35 g (extract) 0.87 g (Chlor. acid)
*Silybum marianum*, e.s. tit. 80% silymarin	Seed	8 g (extract) 6.4 g (silymarin)
*Taraxacum officinale* e.s. tit. 2% inulin	Root	10 g (extract) 0.2 g (inulin)
*Curcuma Longa* e.s. tit. 95% curcumin	Rhizome	10 g (powder) 9.5 g (curcumin)
*Commiphora mukul* Guggul e.s. tit. 10% guggulipids	Resin	15 g (extract) 1.5 g (guggulipids)

e.s. tit.: Dry extract titrated; Chlor. acid: Chlorogenic acid.

## Supplementary Materials

The following are available online at http://www.mdpi.com/2072-6643/9/5/492/s1: Table S1: List of the 84 genes involved in the NAFLD pathogenesis pathway in the array used.
